# In Vitro Activity of Antifungals in Combination With Meropenem or Daptomycin Against Dual *Candida albicans*–Bacterial Biofilms

**DOI:** 10.1111/apm.70047

**Published:** 2025-07-15

**Authors:** Agim Osmani, Sema Askın Keceli, Doganhan Kadir Er, Huseyin Uzuner, Feriha Ercan, Ayca Karagoz Koroğlu

**Affiliations:** ^1^ Department of Medical Microbiology Kocaeli University Faculty of Medicine Kocaeli Turkey; ^2^ Department of Molecular Gastroenterology and Hepatology Institute of Gastroenterology and Hepatology, Kocaeli University Kocaeli Turkey; ^3^ Department of Medical Services and Techniques Vocational School of Health Services, Kocaeli University Kocaeli Turkey; ^4^ Department of Histology and Embryology Marmara University Faculty of Medicine Istanbul Turkey

**Keywords:** antifungals, *C. albicans* biofilm, daptomycin, meropenem, *P. aeruginosa*, *S. epidermidis*

## Abstract

The objective was to investigate the effectiveness of anidulafungin (ANI), voriconazole (VOR), and amphotericin (AMB) combined with meropenem against dual biofilms of *Candida albicans* and *Pseudomonas aeruginosa* , as well as with daptomycin against dual biofilms of *C. albicans* – *Staphylococcus epidermidis* . MIC values of antifungals were determined by CLSI M27‐A3 method. The effectiveness of antifungals in combination with antibiotics against biofilms was tested by Checkerboard analysis. Biofilm quantifications were tested by the XTT method. The biofilm images were captured using SEM. The quantity of *C. albicans*–*S. epidermidis and C. albicans*–*P. aeruginosa* was lower (83.2% and 56.3%, respectively) than that of single Candida biofilm. The SMIC50 values for single *C. albicans* biofilms were 32‐fold higher for AMB and VOR, and 128‐fold higher for ANI when compared to dual Candida biofilms. Daptomycin combined with ANI or VOR showed antagonistic effects against *C. albicans*–*S. epidermidis* biofilms, while meropenem in combination with all antifungals was found to be additive against *C. albicans*–*P. aeruginosa* biofilms. Antifungals and their combinations resulted in a decrease in blastospore and bacterial density, as observed through SEM. In *C. albicans* – *P. aeruginosa* biofilms, using meropenem alongside antifungals would be beneficial due to its additive effect. Daptomycin combined with ANI or VOR may not be effective in *C. albicans*–*S. epidermidis* coinfections.

## 1 Introduction

Candida infections, which demonstrate an increasing incidence worldwide, are the most frequently observed fungal infections. Candida is the third most commonly isolated micro‐organism from bloodstream infections in hospitalized patients [1]. Biofilm formation is one of the most important virulence factors of *Candida* infections. Indeed, most infections by this pathogen are associated with the formation of biofilms on the surfaces of hosts or medical devices, causing high morbidity and mortality. Significantly, biofilms formed by *Candida albicans* are inherently tolerant to antimicrobial therapy, so the susceptibility of *C. albicans* biofilms to current therapeutic agents remains low. Candida biofilm infections may result in invasive fungal infections with a high mortality risk if treatment is not successful [2] and in cases of septicemia, especially in immunosuppressed patients [3, 4].

Invasive candidiasis can be due to the rupture of the host barrier (e.g., severe burns), neutropenia, cancer, and acquired immunodeficiency syndrome. Other factors can contribute to invasive candidiasis, such as invasive medical procedures, catheters, total parenteral nutrition, mechanical ventilation, prolonged hospitalization, treatment with steroids, hyperglycemia, use of broad‐spectrum antibiotics for long periods, and intake of subinhibitory concentrations of antifungals [5, 6].

*Candida albicans* is the most common cause of invasive candidiasis mostly seen in febrile neutropenic patients. The prolonged use of antifungal drugs by patients results in the selection of resistant *C. albicans* strains, making them less susceptible to drugs [7]. Several agents have been tried to increase the efficacy of antifungal drugs against *C. albicans* biofilm. The synergistic effect of cyclosporin A [8] or the inhibitory effect of flufenamic acid [9] in *C. albicans* biofilm when combined with antifungals was demonstrated previously.

The most common symptom of febrile neutropenic patients with invasive candidiasis is fever. It is tough to distinguish between the clinical signs and symptoms of bacterial sepsis and invasive candidiasis in those patients, as both may present with fever or other signs of sepsis [10]. Therefore, these patients are treated simultaneously with broad‐spectrum antibiotics against bacterial infections and antifungals against invasive candidiasis.

Dual therapy with antibacterial and antifungal agents is usually the treatment of choice in cases of *Candida* and bacteria multi‐species biofilm infections. Targeting *C. albicans* in dual‐species biofilms with liposomal amphotericin B has been shown to reduce *Staphylococcus aureus* and methicillin‐resistant *S. aureus* [11]. Thus, there is a need to develop a new fungicidal antifungal agent, probably more effective in dual biofilm infections. Antibiotic resistance could be a problem in the treatment of *C. albicans* ‐bacteria dual biofilms.

The most common type of dual biofilms observed in literature may be given as *C. albicans* – *Pseudomonas aeruginosa* and *C. albicans* – *Staphylococcus epidermidis* biofilms [12]. Dual biofilms of *C. albicans*– *P. aeruginosa* can be seen in conjunction with invasive candidiasis. Although it is known that *P. aeruginosa* inhibits *C. albicans* biofilm development [13], the effect of antifungal and antibiotic treatment and their interactions in infections where dual biofilms are formed has not been fully clarified [14]. In cases where dual *C. albicans* – *P. aeruginosa* biofilm is present, meropenem may be preferred as a broad‐spectrum antibiotic. There are very few studies about the meropenem interaction with antifungals. Previous in vitro and in vivo studies showed that caspofungin, an echinocandin derivative antifungal, had a synergistic effect when applied in combination with meropenem [15]. In a study examining *S. epidermidis* and *C. albicans* dual biofilms, it was found that antifungals had lower efficacy [16] and the treatment using vancomycin in addition to fluconazole was more effective than using fluconazole alone. In addition, it was also observed that daptomycin was found to be more effective than vancomycin in *S. epidermidis* biofilm‐mediated catheter infections [17]. Therefore, it is crucial which antibiotic would lead to a better outcome in patients with combined bacterial and Candida infections when used in combination with antifungals at the same time. To the best of our knowledge, this is the first report testing the efficacy of meropenem and daptomycin against *C. albicans* dual biofilms with *P. aeruginosa* and *S. epidermidis* , respectively. In this study, the efficacy of amphotericin B, voriconazole, and anidulafungin against the single *C. albicans* biofilm or dual biofilm with *S. epidermidis* or *P. aeruginosa* , as well as the efficacy of antifungal‐daptomycin combination against *C. albicans* – *S. epidermidis* biofilm and antifungal‐meropenem combination against *C. albicans* – *P. aeruginosa* biofilm was investigated.

## 2 Materials and Methods

### 2.1 Strains and Growth Conditions

In our study, American Type Culture Collection (ATCC) reference strains *S. epidermidis* ATCC 35984, *P. aeruginosa* ATCC 27853, and *C. albicans* ATCC 90028 were used. The stock cultures of these strains were stored at −80°C. *S. epidermidis* and *P. aeruginosa* were cultured in 10 mL of Tryptic Soy Broth (Merck, Germany) and incubated at 37°C for 24 h, and *C. albicans* was cultured in 10 mL of Sabouraud Dextrose Broth (Merck, Germany) and incubated at 37°C for 48 h. After the incubation period, the *C. albicans* strain was inoculated on Sabouraud Dextrose Agar (SDA) (Merck, Germany), *S. epidermidis* on Sheep Blood Agar (Salubris, Turkey), and *P. aeruginosa* on Mueller‐Hinton Agar (Merck, Germany). For the isolation and enrichment of the strains, a loopful of strains grown in solid media was taken, and the strains were placed in flasks containing 20 mL of liquid medium. The flasks were incubated overnight at 30°C using a 175‐rpm orbital shaker (Labnet, USA). Microorganisms were taken from the flask and transferred to 50 mL vials and centrifuged. After centrifugation, 20 mL of PBS (Sigma‐Aldrich, Germany) was added to the precipitates and washed three times. Then, PBS was removed and 20 mL of medium was added to the precipitates, pipetting was performed to ensure homogenization, and the necessary suspensions were prepared for each microorganism with the use of a spectrophotometer (Shimadzu, Japan).

### 2.2 Antifungals and Antibiotics

The stock concentrations of amphotericin B (AMB), voriconazole (VOR) and anidulafungin (ANI) were prepared as 5120 μg/mL in dimethyl sulfoxide. The concentration ranges used in this study were as follows: 0.015–32 μg/mL for AMB (Sigma‐Aldrich, Germany), 0.005–16 μg/mL for VOR (Sigma‐Aldrich, Germany), and 0.005–16 μg/mL for ANI (Pfizer, Germany). As antibacterial agents, daptomycin (0.125–64 μg/mL) and meropenem (0.125–64 μg/mL) (Sigma‐Aldrich, Germany) were used in active powder form and dissolved in sterile water.

### 2.3 Single and Dual Biofilm Formation

Single biofilm of *C. albicans* and dual biofilms of *C. albicans* – *S. epidermidis* and *C. albicans* – *P. aeruginosa* were formed using the following method: Suspensions of each microorganism were prepared using a spectrophotometer. *C. albicans* suspension was prepared in sterile RPMI‐1640 medium (Roswell Park Memorial Institute) (Sigma‐Aldrich, Germany), with a pH of 7.0 at a density of 1.0 × 10^6^ cells/mL [18]. For *S. epidermidis* and *P. aeruginosa* , inoculum was performed at a density of 1.0 × 10^7^ cells/mL in Brain Heart Infusion (BHI) medium [19, 20]. To form single biofilms from inoculum suspensions, 100 μL of each microorganism suspension was placed on 96‐well flat‐bottom ELISA plates (Corning, USA). To create dual biofilms, 100 μL of both *C. albicans* and the bacterial suspensions were placed in the same well. Thus, in the case of dual biofilms, the wells contained a total of 200 μL Candida and bacterial suspensions in RPMI‐1640 and BHI medium mixture.

Then, ELISA plates for both single and dual biofilms were incubated at 37°C for 24 h. After the biofilm formation, the liquid in the upper part of the wells was carefully aspirated without disturbing the biofilms. Each well was then washed three times with 200 μL of sterile PBS. Finally, the biofilms at the bottom of the wells were quantified.

### 2.4 Quantification of Single and Dual Biofilms

Biofilm quantification was performed using the colorimetric XTT (2,3‐bis (2‐methoxy‐4‐nitro‐5‐sulphophenyl)‐2H‐tetrazolium) (Sigma‐Aldrich, Germany) method that measures the metabolic activity of the cells [14]. XTT was used to determine the biofilm amount at the bottom of the wells, and biofilm inhibition was also measured with XTT by adding an antimicrobial agent (antifungal/antibiotic) to the biofilm. In both single and dual biofilms, one of the biofilm wells without antifungal or antibiotic was used as a positive control for each microorganism, and one well of medium containing XTT was used as a negative control.

Menadione (Sigma‐Aldrich, Germany) was prepared as a 10 mM stock solution, and 100 μL XTT/menadione was dispensed into all pre‐washed wells containing biofilm, including positive and negative controls. The plates were covered with aluminum foil and incubated for 5 h at 37°C; then, the formation of brown formazan product as a result of the reduction of XTT was observed. After incubation, optical density (OD) values were obtained at a wavelength of 490 nm using a plate reader (Alisei, Italy). The experiments with XTT were repeated three times.

The XTT method was used to measure *C. albicans* , *S. epidermidis* , and *P. aeruginosa* single biofilms and also dual *C. albicans* – *S. epidermidis* and *C. albicans* – *P. aeruginosa* biofilms, and to detect biofilm quantity after the addition of antifungal alone or antifungal‐antibiotic combinations against *C. albicans* biofilm, *C. albicans*– *S. epidermidis* , and *C. albicans* – *P. aeruginosa* dual biofilms.

### 2.5 Antifungal Susceptibility Tests

Antifungal susceptibility tests were conducted following the CLSI M27‐A3 guidelines. This method allowed us to determine the minimum inhibitory concentrations (MIC) for the planktonic phase, before biofilm formation. The MIC values were established as follows: for VOR and ANI, the MIC was identified as the lowest concentration that resulted in at least an 80% reduction in turbidity compared to the control well. For AMB, the MIC was defined as the concentration at which no growth was observed.

### 2.6 Microdilution Checkerboard Method

The efficacy of (1) AMB and daptomycin, (2) VOR and daptomycin, (3) ANI and daptomycin on dual biofilms formed by *C. albicans* and *S. epidermidis* and the efficacy of (1) AMB and meropenem, (2) VOR and meropenem, (3) ANI and meropenem on dual biofilms formed by *C. albicans* and *P. aeruginosa* were investigated using the microdilution checkerboard method. Fractional inhibitory concentration index (FICi) was calculated for each antimicrobial according to the following formula:

$$\begin{array}{c} \text{Fractional inhibitory concentration index}\;\left(\text{FICi}\right)=\text{ΣFICa}+b\\ =\text{FICa}+\text{FICb} \end{array}$$

The values of FICi were considered as follows: If FICi ≤ 0.5, synergistic; If FICi = 0.5–1 additive (additional effect), If FICi = 1–4 indifferent, and If FICi > 4 antagonistic [20]. After biofilms were formed, the sessile MICs (SMIC, the MIC value of biofilm) were determined in microplates as described previously [21, 22]. For each microorganism, 100 μL of the suspension was placed into wells, 12 wells of the same suspension were used for the positive control, and a well containing only RPMI medium was used as the negative control.

Plates were incubated at 37°C for 24 h. Then the suspensions in the wells were removed and the wells were washed three times with 200 μL of PBS. Of 100 μL serial dilutions of RPMI containing biofilm were added to the wells, containing antifungal and daptomycin or meropenem as described above. Then, 100 μL of RPMI was added to wells without antifungal and antibiotic (meropenem or daptomycin) and to the negative control well. Microplates were incubated at 37°C for 24 h. After incubation, the plates were washed three times with PBS. Then, 100 μL of XTT/menadione mixture was added to each well and the plates were incubated for 5 h.

The absorbance measurement of the microplates was performed at 490 nm using a plate reader. The lowest concentration caused a 50% and 80% decrease in absorbance compared to the control well (only microorganism suspension) for azoles and ANI, and %100 reductions for AMB was determined as the sessile MIC (SMIC).

### 2.7 Examination of Single and Dual Biofilms by Scanning Electron Microscopy

To perform Scanning Electron Microscopy (SEM) examination, after 24 h biofilm formation, the biofilm suspension in the wells was transferred to small square coverslips suitable for SEM. After 24 h, the coverslips were washed with PBS three times to remove planktonic cells. Coverslips were fixed in 2.5% glutaraldehyde (Sigma‐Aldrich, Germany) for 2 h and liquid osmium tetroxide (Sigma‐Aldrich, Germany) for 1 h and left to air dry. The coverslips were incubated in 35%, 50%, 70%, and 95% ethanol for 10 min periods, then dehydrated for 10 min incubation in 100% ethanol and allowed to air dry again for 20 min. SEM coverslips were mounted and coated with gold/palladium [23, 24]. Imaging of the samples was performed in SEM (JEOL JSM‐5200, Japan) high‐vacuum mode at a voltage of 15 kV and with magnifications of 500×, 1500×, and 3500×.

### 2.8 Statistical Evaluation

The statistical data analysis was conducted using the GraphPad (Prism version 6.01) program. The data were analyzed using a *t*‐test to verify statistical significance, with a *p*‐value of < 0.05 considered significant.

## 3 Results

### 3.1 Single and Dual Biofilm Quantification

**FIGURE 1:**
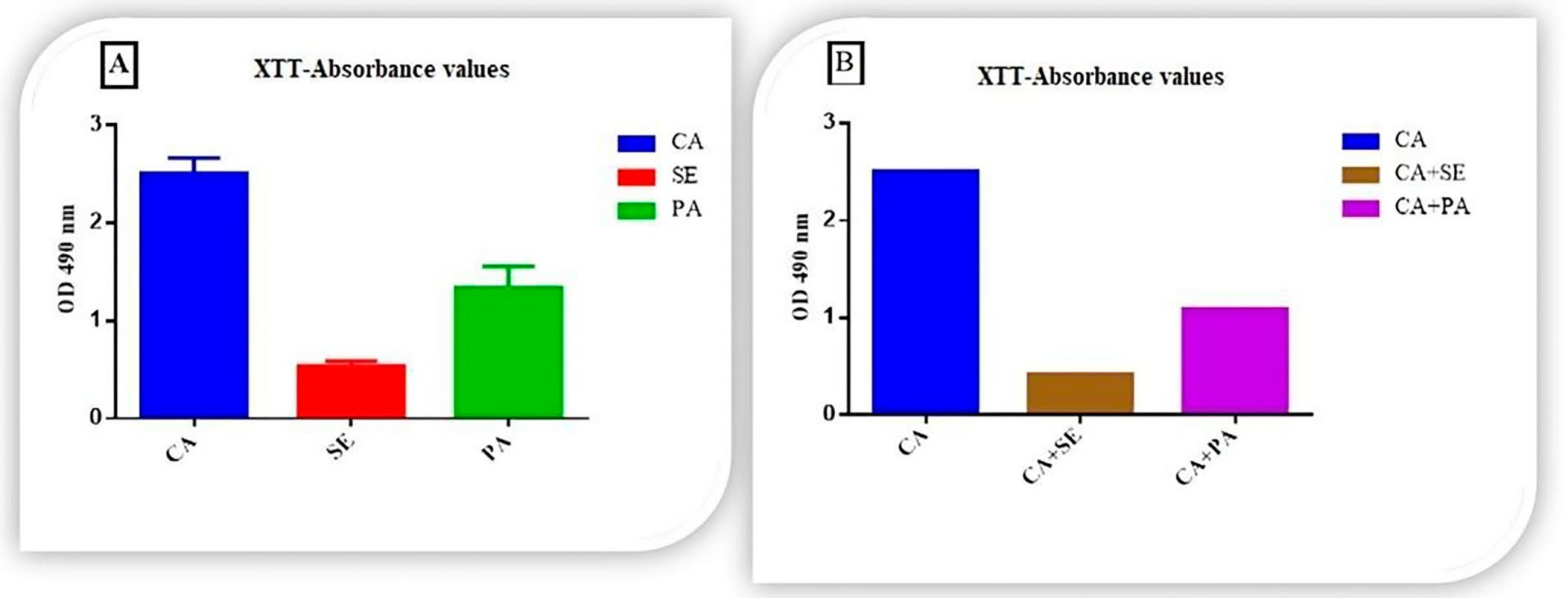
Biofilm absorbance values were determined by XTT method. (A) Single biofilm absorbance values, CA = *C. albicans* ; SE = *S. epidermidis* ; PA = *P. aeruginosa* . (B) Comparison of the absorbance values of the biofilm formed by *C. albicans* alone with the absorbance values of dual biofilms formed by *C. albicans* – *S. epidermidis* and *C. albicans* ‐ *P. aeruginosa* .

The optical density (OD) values of biofilms formed by single species of *C. albicans, P. aeruginosa*, and *S. epidermidis* , as well as dual biofilms composed of *C. albicans* and two different bacteria, were measured using the XTT method and are illustrated in Figure 1.

Upon evaluation of the OD values for the single biofilms, *C. albicans* exhibited the highest quantity (OD: 2.512), followed by *P. aeruginosa* (OD: 1.342), while *S. epidermidis* had the lowest value (OD: 0.538). In comparison to the single *C. albicans* biofilm, the dual biofilm of *C. albicans* – *S. epidermidis* showed an 83.2% reduction in OD (OD: 0.420), with a statistically significant difference between the two values (*p* < 0.001). Similarly, the dual biofilm of *C. albicans‐P. aeruginosa* demonstrated a 56.3% decrease in OD (OD: 1.092) compared to the single *C. albicans* biofilm, also with a statistically significant difference (*p* < 0.001).

### 3.2 Antifungal Susceptibilities of *C. albicans* Biofilm

**FIGURE 2:**
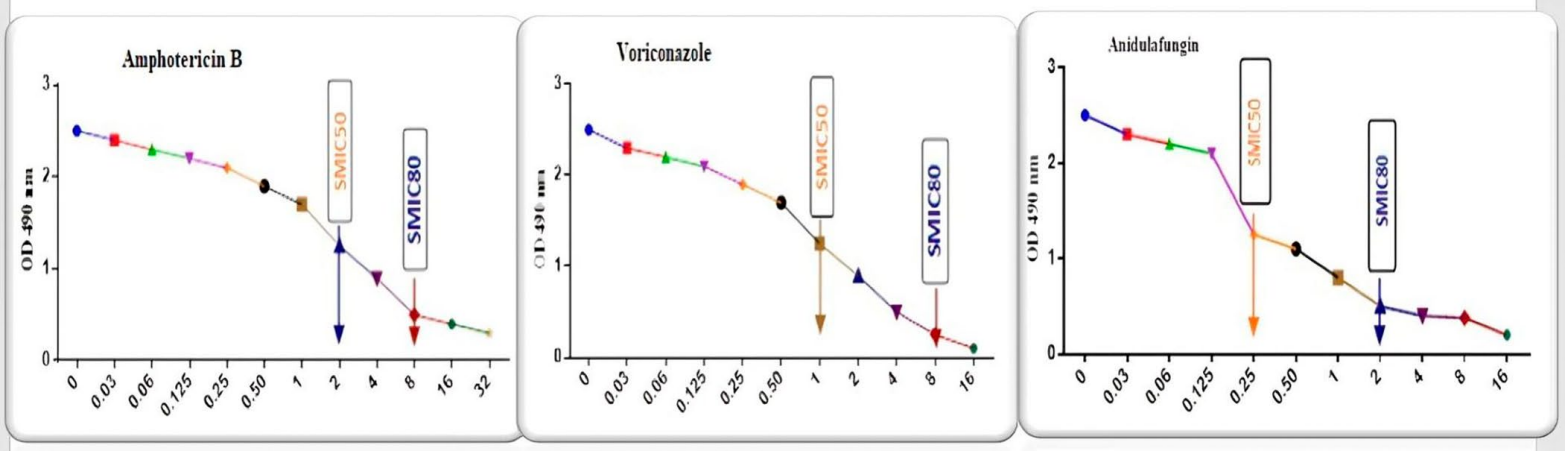
Amphotericin B, voriconazole and anidulafungin susceptibility of *C. albicans* biofilm, SMIC50 and SMIC80 values.

The planktonic MIC values of *C. albicans* against antifungals and the MIC values of bacteria against antibiotics were given as Table S1, and all were found to be susceptible. When the efficacy of AMB, VOR, and ANI on single *C. albicans* biofilm was tested after 24 h, SMIC50 values were determined to be 2, 1, and 0.25 μg/mL, respectively; and SMIC80 values were 8, 8, and 2 μg/mL, respectively. In comparison to the planktonic MIC values, SMIC80 values were 16, 32, and 8 fold higher in AMB, VOR, and ANI, respectively; SMIC50 values were 4 fold higher for AMB and VOR, whereas the two values were equal for ANI (Figure 2).

### 3.3 Antifungal Susceptibilities of Dual *C. albicans* – *S. epidermidis* and *C. albicans‐P. aeruginosa* Biofilms

For AMB, VOR, and ANI, the SMIC50 values of the *C. albicans* – *S. epidermidis* dual biofilm were 64, 32, and 32 μg/mL, respectively. Whereas, the SMIC50 values of the *C. albicans‐P. aeruginosa* biofilm were 8 μg/mL for all antifungals tested. Compared to the SMIC50 values of the single *C. albicans* biofilm, the SMIC50 values of the dual biofilm were 32‐fold higher for AMB and VOR and 128‐fold higher for ANI.

### 3.4 Antifungal‐Antibiotic Combination Susceptibilities in Dual Biofilms

#### 3.4.1 *C. albicans* – *S. epidermidis* Biofilm

When the combinations of daptomycin with all antifungals were tested, SMIC50 (μg/mL) values and FIC values were determined. It was found that daptomycin had an indifferent effect when used in combination with AMB, and an antagonistic effect when used in combination with VOR or ANI (Table 1).

**TABLE 1:** Results of checkerboard analysis for SMIC50 values when daptomycin and meropenem are combined with amphotericin B, voriconazole and anidulafungin in dual biofilms.

Dual biofilms	MIC values when antifungals combined with daptomycin		
	SMIC50 (μg/mL)	ΣFIC^a^ (μg/mL)	Efficacy
*C. albicans* + *S. epidermidis* biofilm			
Amphotericin B	128 μg/mL	4	Antagonistic
Voriconazole	128 μg/mL	6	Antagonistic
Anidulafungin	128 μg/mL	6	Indifferent
*C. albicans* + *P. aeruginosa* biofilm	**MIC values when antifungals combined with meropenem**		
Amphotericin B	4	1	Additive
Voriconazole	4	1	Additive
Anidulafungin	4	1	Additive

^a^ Fractional inhibitory concentration (FIC) index.

#### 3.4.2 *C. albicans‐P. aeruginosa* Biofilm

Data demonstrating the susceptibility of *C. albicans‐P. aeruginosa* biofilm to the antifungal‐meropenem combination is presented in Table 1. As a result, it was determined that meropenem had an additive effect when combined with AMB, VOR, and ANI.

### 3.5 Scanning Electron Microscopy Examination

**FIGURE 3:**
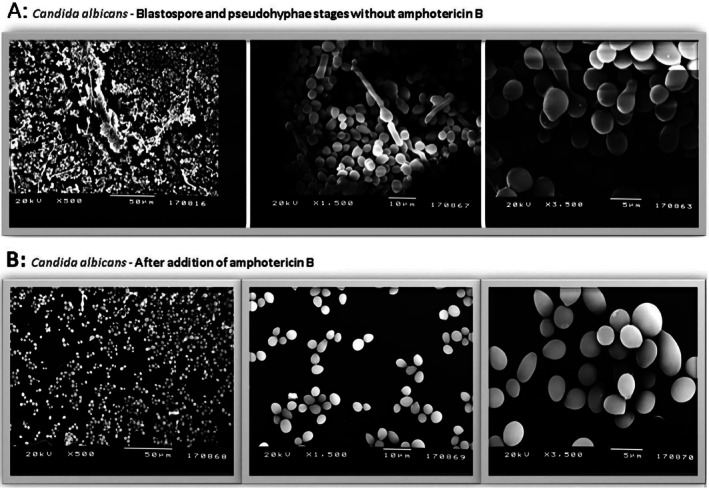
(A) *C. albicans* biofilm without amphotericin B. (B) The effect of amphotericin B on *C. albicans* biofilm.

The images of *C. albicans* biofilm at different magnifications after 48 h of incubation are shown in Figure 3. Blastospore and pseudohyphae formation can be seen in images taken without AMB (Figure 3A). After the addition of AMB, no pseudohyphae and a decrease in blastospore density, that is, biofilm density, were observed (Figure 3B).

**FIGURE 4:**
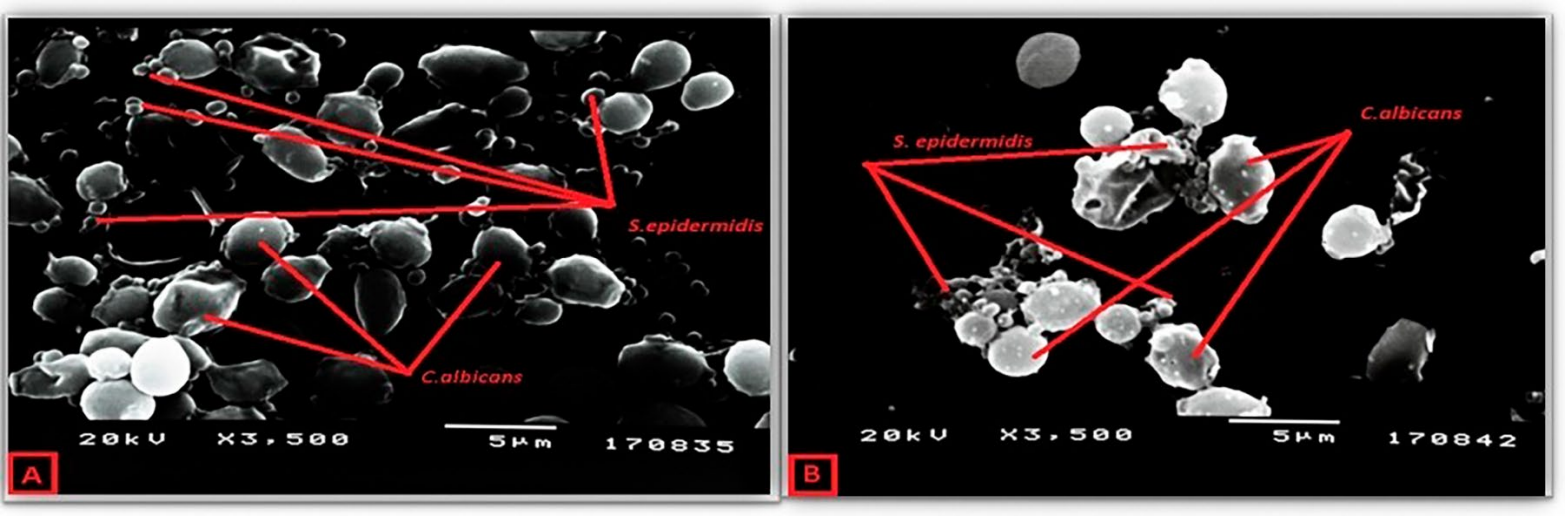
Imaging of *C. albicans* – *S. epidermidis* dual biofilm after 24 h of incubation. (A) *C. albicans* – *S. epidermidis* dual biofilm without the addition of amphotericin B and daptomycin. (B) The effect of amphotericin B and daptomycin combination on *C. albicans* – *S. epidermidis* dual biofilm.

**FIGURE 5:**
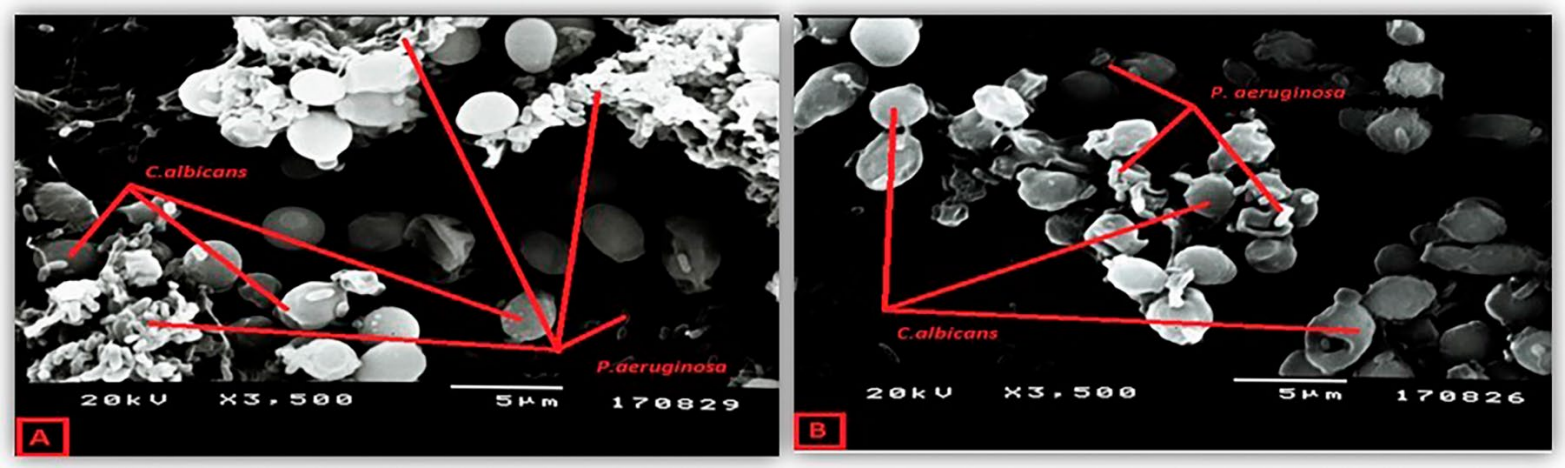
Imaging of *C. albicans* ‐ *P. aeruginosa* dual biofilm after 24 h of incubation. (A) *C. albicans* ‐ *P. aeruginosa* dual biofilm without the addition of amphotericin B and meropenem. (B) The effect of amphotericin B and meropenem combination on *C. albicans‐P. aeruginosa* dual biofilm.

The effect of the combination of AMB and daptomycin on the *C. albicans* – *S. epidermidis* dual biofilm (Figure 4A,B) and the effect of the combination of AMB and meropenem on *C. albicans* – *P. aeruginosa* biofilm were evaluated. A significant decrease in bacterial density was observed, whereas a little decrease in blastospores was observed with combined antifungal and antimicrobial administration (Figure 5A,B).

## 4 Discussion

In this study, the quantity of single *C. albicans* biofilm and dual biofilm with *S. epidermidis* and *P. aeruginosa* was evaluated. In addition, the effect of the combination of daptomycin with antifungals on *C. albicans*– *S. epidermidis* and the effect of meropenem in combination with antifungals against *C. albicans* – *P. aeruginosa* biofilm were studied. There are many studies evaluating the effects of polymicrobial bacterial plus *C. albicans* biofilm. In a study, it was shown that *C. albicans* biofilm formation was reduced when coexisting with a bacterial biofilm. It has been suggested that this effect was caused by the suppression of genes associated with the hyphae transition of *C. albicans* and bacterial particles negatively affecting the biofilm architecture. Compared with the rate of biofilm formation when *C. albicans* was incubated alone, it was found that biofilm formation of *C. albicans* decreased when incubated with each of six different bacteria (*Escherichia coli, P. aeruginosa, Proteus vulgaris, Staphylococcus aureus, Streptococcus pyogenes*, and *S. salivarius* ).

Evaluating biofilm quantification based on absorbance values in this study showed that the dual biofilm quantity of *C. albicans‐P. aeruginosa* was reduced by 56.3% compared to single *C. albicans* biofilm. *P. aeruginosa* appeared to inhibit the formation of biofilms by *C. albicans* when both were incubated together. This finding is consistent with research indicating that *P. aeruginosa* can suppress chlamydospore and hyphae formation in *C. albicans* . It has been suggested that bacteria contribute to this inhibition not only through metabolites produced during their growth phases but also by inducing structural changes in *C. albicans* biofilm formation [25]. Similarly, the dual biofilm of *C. albicans* – *S. epidermidis* led to an 83.2% reduction in biofilm quantity compared to the biofilm formed by *C. albicans* alone. These results indicate that *S. epidermidis* significantly inhibits *C. albicans* biofilm formation.

In a study by Jacobson et al., MIC values for ANI were found to be ≤ 2 μg/mL against 30 planktonic *C. albicans* isolates, and the biofilm SMIC value range for ANI was ≤ 0.03–16 μg/mL. Shuford et al. found the planktonic MIC90 values of AMB, VOR, and CASP as 0.25, 0.06, and 0.5 μg/mL, respectively [22]. In our study, SMIC50 values in *C. albicans* biofilm were found 4 times higher against AMB and VOR compared to planktonic cells, while the two values were equal for ANI. The SMIC80 values were 16, 32, and 8 times higher for AMB, VOR, and ANI, respectively. Since the SMIC50 values of ANI did not change and the SMIC80 value was only 8 times higher in dual biofilms, these results suggested that ANI may be more effective than other antifungal agents.

In a study, the effects of fluconazole as an antifungal and vancomycin as an antibiotic against *C. albicans* – *S. epidermidis* dual biofilm were investigated. Two strains of *S. epidermidis* (a slime‐producing wild‐type RP62A strain and a slime‐negative mutant M7 strain) were used in that study, and the extracellular polymer produced by wild‐type *S. epidermidis* RP62A was shown to inhibit fluconazole penetration in mixed fungal‐bacterial biofilms. The presence of *C. albicans* in dual biofilms was observed to protect the *S. epidermidis* M7 strain against vancomycin. These results showed that fungal cells can alter the effect of antibiotics, and bacteria can affect the antifungal activity on dual fungal‐bacterial biofilms [16]. These results were also supported by findings of our study by high MIC values detected for antifungals in *C. albicans* – *S. epidermidis* biofilm.

In a study by Stewart et al., the penetration rate of daptomycin into *S. epidermidis* biofilm was measured, and it was observed that fluorescent‐labeled daptomycin could easily penetrate thick *S. epidermidis* biofilms, and the penetration took place in a very short time [17]. In our study, an indifferent effect was found for the combination of daptomycin and AMB in the *C. albicans* – *S. epidermidis* biofilm, and an antagonist effect for the combination of VOR and ANI. Although in our study, daptomycin has been shown to be effective alone on *S. epidermidis* biofilm, the indifferent or antagonistic effect on *C. albicans* – *S. epidermidis* dual biofilm suggests that both microorganisms might form a protective shield and reduce the antimicrobial effect. It has been shown that cell wall polysaccharides specific to *C. albicans* rapidly coat *S. aureus* cells, and this may provide additional protection by preventing the antibiotic from reaching its cellular target [26].

*Candida albicans* and *P. aeruginosa* can be isolated together at various sites of infection, including burn wounds, contaminated catheters, and the lungs, and they can also affect the virulence of each other [27]. According to the MIC values of *C. albicans* and *P. aeruginosa* against antifungals in our study, meropenem demonstrated an additive effect when added to antifungals. Therefore, meropenem may increase the antifungal effect when antibiotic treatment is required in addition to antifungal treatment of *C. albicans* and *P. aeruginosa* coinfections. A synergistic effect of CASP and meropenem was shown in the treatment of an in vivo candidiasis model. This synergy was tested by checkerboard analysis of antimicrobial susceptibility in vitro and the degree of inflammation in vivo. In conclusion, it was shown that concomitant CASP and meropenem treatment may be beneficial [15]. Similarly, in our study, an additive effect was also found in the *C. albicans* – *P. aeruginosa* dual biofilm when ANI and meropenem were combined, which may be considered an important finding to increase treatment efficacy.

A study by Rodrigues et al. assessed the polymicrobial nature of ventilator‐associated pneumonia (VAP), characterized polymicrobial bacterial‐fungal biofilms that colonize the endotracheal tube surface in VAP, and evaluated the combination of polymyxin B (Poly B) and AMB treatment. They noted that this combination has a potentially synergistic therapeutic effect against *P. aeruginosa‐C. albicans* dual biofilm. However, only a high dose (256 μg/mL) of Poly B was able to eliminate single and dual biofilms. After antimicrobial treatment, *P. aeruginosa* was inhibited at 2 h., while the inhibiting effect on *C. albicans* began after 14 h [20]. In our study, when AMB was used together with meropenem in *C. albicans* and *P. aeruginosa* biofilms, an additive effect was found even if they are used in low concentration (4 μg/mL), in contrast to Rodrigues et al.'s study.

In a study by Alam et al., *C. albicans* was shown to increase *P. aeruginosa* tolerance to meropenem at a clinically relevant concentration of 5 μg/mL. This effect is biofilm‐specific and dependent on *C. albicans* extracellular matrix polysaccharides, mannan and glucan. It is thought that mannan and glucan, which are fungal cell wall components, are secreted into the extracellular matrix of the *C. albicans* ‐ *P. aeruginosa* dual biofilm, and this plays an important role in increasing the tolerance of *P. aeruginosa* to meropenem. Therefore, it has been suggested that a possible increase in tolerance should be considered if meropenem is included in the treatment protocol of coinfected patients. Another finding is that the secreted *C. albicans* extracellular matrix polysaccharides protect *P. aeruginosa* by reducing the efficacy of meropenem. Persistent bacterial infections may occur due to the structural characteristics of protected cells. This can lead to the development of true resistance as a result of continuous exposure to sub‐minimum inhibitory concentration (sub‐MIC) antibiotic levels. These findings highlight the importance of early diagnosis of dual biofilm infections. It was concluded that more effective treatment options, such as antibiotic‐antifungal combination therapy, should be considered [28].

## 5 Conclusion

It is crucial to explore alternative strategies to overcome the limitations of current therapies against fungal infections associated with biofilms. In this respect, recent analyses of gene‐drug interactions suggest that the focus on the development of antifungals targeting specific pathogens can lead to more potent and effective therapies, especially in treating invasive fungal infections.

The novelty of this study is to test the efficacy of daptomycin and meropenem when used with antifungals against dual biofilms of *C. albicans*–*S*. *epidermidis* and *C. albicans‐P. aeruginosa*, respectively. The efficacy of the combination of AMB and daptomycin showed no significant difference. However, when daptomycin was combined with VOR and ANI, it exhibited an antagonistic effect on the dual biofilm of *C. albicans*– *S. epidermidis* . Therefore, using daptomycin alongside these antifungals may not be the best option for treatment protocols in such cases.

In the dual biofilm of *C. albicans* – *P. aeruginosa* , an additive effect of meropenem was observed when combined with all antifungals tested. If a broad‐spectrum antibiotic is to be added to an antifungal treatment protocol for polymicrobial infections involving *C. albicans* and *P. aeruginosa* , it is essential to consider the additive effect of meropenem. Although all observations were based on in vitro test results and should be confirmed by in vivo tests, the additive effect of meropenem may assist clinicians in planning an antibiotic regimen with concurrent antifungal use.

## Conflicts of Interest

The authors declare no conflicts of interest.

## Supporting information


**Table S1.** The planctonic MIC values of antifungals against *C. albicans* and MIC values of antibiotics against *S. epidermidis* and/or *P. aeruginosa* .

## Data Availability

The data that support the findings of this study are available from the corresponding author upon reasonable request.

## References

[1] B. E. Shields , M. Rosenbach , Z. Brown‐Joel , A. P. Berger , B. A. Ford , and K. A. Wanat , “Angioinvasive Fungal Infections Impacting the Skin: Background, Epidemiology, and Clinical Presentation,” Journal of the American Academy of Dermatology 80, no. 4 (2019): 869–880.e5, 10.1016/j.jaad.2018.04.059. 30102951

[2] H. T. Taff , J. E. Nett , R. Zarnowski , et al., “A Candida Biofilm‐Induced Pathway for Matrix Glucan Delivery: Implications for Drug Resistance,” PLoS Pathogens 8, no. 8 (2012): e1002848, 10.1371/journal.ppat.1002848. 22876186 PMC3410897

[3] S. Nami , R. Mohammadi , M. Vakili , K. Khezripour , H. Mirzaei , and H. Morovati , “Fungal Vaccines, Mechanism of Actions and Immunology: A Comprehensive Review,” Biomedicine & Pharmacotherapy 109 (2019): 333–344, 10.1016/j.biopha.2018.10.075. 30399567

[4] T. Sakagami , T. Kawano , K. Yamashita , et al., “Antifungal Susceptibility Trend and Analysis of Resistance Mechanism for Candida Species Isolated From Bloodstream at a Japanese University Hospital,” Journal of Infection and Chemotherapy 25, no. 1 (2019): 34–40, 10.1016/j.jiac.2018.10.007. 30401513

[5] M. A. Pfaller and D. J. Diekema , “Epidemiology of Invasive Candidiasis: A Persistent Public Health Problem,” Clinical Microbiology Reviews 20, no. 1 (2007): 133–163, 10.1128/cmr.00029-06. 17223626 PMC1797637

[6] J. F. Ha , C. M. Italiano , C. H. Heath , S. Shih , S. Rea , and F. M. Wood , “Candidemia and Invasive Candidiasis: A Review of the Literature for the Burns Surgeon,” Burns 37, no. 2 (2011): 181–195. 20395056 10.1016/j.burns.2010.01.005

[7] C. C. Dawson , C. Intapa , and M. A. Jabra‐Rizk , “‘Persisters’: Survival at the Cellular Level,” PLoS Pathogens 7, no. 7 (2011): e1002121, 10.1371/journal.ppat.1002121. 21829345 PMC3145784

[8] R. B. Shinde , N. M. Chauhan , J. S. Raut , and S. M. Karuppayil , “Sensitization of *Candida albicans* Biofilms to Various Antifungal Drugs by Cyclosporine A,” Annals of Clinical Microbiology and Antimicrobials 11 (2012): 1–7. 23035934 10.1186/1476-0711-11-27 PMC3508915

[9] A. A. Chavez‐Dozal , M. Jahng , H. S. Rane , et al., “ *In Vitro* Analysis of Flufenamic Acid Activity Against *Candida albicans* Biofilms,” International Journal of Antimicrobial Agents 43, no. 1 (2014): 86–91. 24156913 10.1016/j.ijantimicag.2013.08.018 PMC3902125

[10] A. L. Colombo , J. N. de Almeida Júnior , M. A. Slavin , S. C. Chen , and T. C. Sorrell , “Candida and Invasive Mould Diseases in Non‐Neutropenic Critically Ill Patients and Patients With Haematological Cancer,” Lancet Infectious Diseases 17, no. 11 (2017): e344–e356. 28774702 10.1016/S1473-3099(17)30304-3

[11] Y. Luo , D. F. McAuley , C. R. Fulton , J. Sá Pessoa , R. McMullan , and F. T. Lundy , “Targeting *Candida albicans* in Dual‐Species Biofilms With Antifungal Treatment Reduces *Staphylococcus Aureus* and MRSA *In Vitro* ,” PLoS One 16, no. 4 (2021): e0249547. 33831044 10.1371/journal.pone.0249547 PMC8031443

[12] M. M. Harriott and M. C. Noverr , “Importance of Candida–Bacterial Polymicrobial Biofilms in Disease,” Trends in Microbiology 19, no. 11 (2011): 557–563. 21855346 10.1016/j.tim.2011.07.004 PMC3205277

[13] H. M. Bandara , J. Y. Yau , R. M. Watt , L. J. Jin , and L. P. Samaranayake , “ *Pseudomonas aeruginosa* Inhibits *In‐Vitro* Candida Biofilm Development,” BMC Microbiology 10, no. 1 (2010): 1–9. 20416106 10.1186/1471-2180-10-125 PMC2874548

[14] S. J. Park , K. H. Han , J. Y. Park , S. J. Choi , and K. H. Lee , “Influence of Bacterial Presence on Biofilm Formation of *Candida albicans* ,” Yonsei Medical Journal 55, no. 2 (2014): 449–458. 24532517 10.3349/ymj.2014.55.2.449 PMC3936627

[15] S. K. Ozcan , F. Budak , A. Willke , S. Filiz , P. Costur , and H. Dalcik , “Efficacies of Caspofungin and a Combination of Caspofungin and Meropenem in the Treatment of Murine Disseminated Candidiasis,” APMIS 114, no. 12 (2006): 829–836. 17207082 10.1111/j.1600-0463.2006.apm_450.x

[16] B. Adam , G. S. Baillie , and L. J. Douglas , “Mixed Species Biofilms of *Candida albicans* and *Staphylococcus epidermidis* ,” Journal of Medical Microbiology 51, no. 4 (2002): 344–349. 11926741 10.1099/0022-1317-51-4-344

[17] P. S. Stewart , W. M. Davison , and J. N. Steenbergen , “Daptomycin Rapidly Penetrates a *Staphylococcus epidermidis* Biofilm,” Antimicrobial Agents and Chemotherapy 53, no. 8 (2009): 3505–3507. 19451285 10.1128/AAC.01728-08 PMC2715639

[18] C. G. Pierce , P. Uppuluri , S. Tummala , and J. L. Lopez‐Ribot , “A 96 Well Microtiter Plate‐Based Method for Monitoring Formation and Antifungal Susceptibility Testing of *Candida albicans* Biofilms,” JoVE Journal of Visualized Experiments 44 (2010): e2287. 10.3791/2287 PMC3185615 21048668

[19] L. Lown , B. M. Peters , C. J. Walraven , M. C. Noverr , and S. A. Lee , “An Optimized Lock Solution Containing Micafungin, Ethanol and Doxycycline Inhibits *Candida albicans* and Mixed *C. albicans* –*Staphyloccoccus aureus* Biofilms,” PLoS One 11, no. 7 (2016): e0159225. 27428310 10.1371/journal.pone.0159225 PMC4948884

[20] M. E. Rodrigues , S. P. Lopes , C. R. Pereira , et al., “Polymicrobial Ventilator‐Associated Pneumonia: Fighting *In Vitro Candida albicans‐Pseudomonas aeruginosa * Biofilms With Antifungal‐Antibacterial Combination Therapy,” PLoS One 12, no. 1 (2017): e0170433. 28114348 10.1371/journal.pone.0170433 PMC5256963

[21] M. C. Arendrup , S. Park , S. Brown , M. Pfaller , and D. S. Perlin , “Evaluation of CLSI M44‐A2 Disk Diffusion and Associated Breakpoint Testing of Caspofungin and Micafungin Using a Well‐Characterized Panel of Wild‐Type and Fks Hot Spot Mutant Candida Isolates,” Antimicrobial Agents and Chemotherapy 55, no. 5 (2011): 1891–1895. 21357293 10.1128/AAC.01373-10 PMC3088242

[22] J. A. Shuford , K. E. Piper , J. M. Steckelberg , and R. Patel , “ *In Vitro* Biofilm Characterization and Activity of Antifungal Agents Alone and in Combination Against Sessile and Planktonic Clinical *Candida albicans* Isolates,” Diagnostic Microbiology and Infectious Disease 57, no. 3 (2007): 277–281. 17141454 10.1016/j.diagmicrobio.2006.09.004

[23] S. E. Fratesi , F. L. Lynch , B. L. Kirkland , and L. R. Brown , “Effects of SEM Preparation Techniques on the Appearance of Bacteria and Biofilms in the Carter Sandstone,” Journal of Sedimentary Research 74, no. 6 (2004): 858–867.

[24] M. M. Harriott and M. C. Noverr , “ *Candida albicans* and *Staphylococcus aureus* Form Polymicrobial Biofilms: Effects on Antimicrobial Resistance,” Antimicrobial Agents and Chemotherapy 53, no. 9 (2009): 3914–3922. 19564370 10.1128/AAC.00657-09 PMC2737866

[25] S. K. Ozcan , D. Dundar , and G. S. Tamer , “Klinik *Pseudomonas aeruginosa* Suşlarının Antikandidal Aktiviteleri ve *İn Vitro* Candida Biyofilm Oluşumunun İnhibisyonu,” Mikrobiyoloji Bülteni 46, no. 1 (2012): 39–46. 22399170

[26] E. F. Kong , C. Tsui , S. Kucharíková , D. Andes , P. Van Dijck , and M. A. Jabra‐Rizk , “Commensal Protection of *Staphylococcus aureus* Against Antimicrobials by *Candida albicans* Biofilm Matrix,” MBio 7, no. 5 (2016): e01365‐16. 27729510 10.1128/mBio.01365-16 PMC5061872

[27] A. Y. Peleg and D. C. Hooper , “Hospital‐Acquired Infections due to Gram‐Negative Bacteria,” New England Journal of Medicine 362, no. 19 (2010): 1804–1813. 20463340 10.1056/NEJMra0904124 PMC3107499

[28] F. Alam , D. Catlow , A. Di Maio , J. M. A. Blair , and R. A. Hall , “ *Candida albicans* Enhances Meropenem Tolerance of *Pseudomonas aeruginosa* in a Dual‐Species Biofilm,” Journal of Antimicrobial Chemotherapy 75, no. 4 (2020): 925–935. 31865379 10.1093/jac/dkz514 PMC7069478

