# Supplementary material for: In Vitro Activity of Antifungals in Combination With Meropenem or Daptomycin Against Dual Candida albicans–Bacterial Biofilms
Source: APMIS. 2025 Jul 15;133(7):e70047. doi: 10.1111/apm.70047 (PMC12263515; doi:10.1111/apm.70047)
Supplement: Supplementary file 1 — Table S1. The planctonic MIC values of antifungals against C. albicans and MIC values of antibiotics against S. epidermidis and/or P. aeruginosa . [file APM-133-0-s001.docx]

|  | **Antimicrobials** | **MIC values (µg/mL)** |
| --- | --- | --- |
| ***C. albicans*** | Amphotericin | 0.5 |
|  | Voriconazole | 0.25 |
|  | Anidulafungin | 0.25 |
|  |  |  |
| ***S. epidermidis*** | Daptomycin | 0.25 |
|  |  |  |
| ***P. aeruginosa*** | Meropenem | 0.5 |

**Table S1.** The planctonic MIC values of antifungals against *C. albicans* and MIC values of antibiotics against *S. epidermidis* and/or *P. aeruginosa*.
